# Custom CARs: Leveraging the Adaptability of Allogeneic CAR Therapies to Address Current Challenges in Relapsed/Refractory DLBCL

**DOI:** 10.3389/fimmu.2022.887866

**Published:** 2022-05-18

**Authors:** Nikeshan Jeyakumar, Melody Smith

**Affiliations:** ^1^ Divisions of Hematology and Oncology, Stanford University School of Medicine, Stanford, CA, United States; ^2^ Division of Blood and Marrow Transplantation and Cellular Therapy, Stanford University School of Medicine, Stanford, CA, United States

**Keywords:** allogeneic CAR T cells, DLBCL - diffuse large B cell lymphoma, adoptive cell immunotherapy, hematopoietic (stem) cell transplantation, GVHD

## Abstract

Cellular therapies have transformed the treatment of relapsed/refractory diffuse large B-cell lymphoma (r/r DLBCL), which typically does not respond well to salvage chemotherapy. Recently, approximately 40% of r/r DLBCL patients across three different trials achieved a complete remission at 1 year after receiving treatment with autologous chimeric antigen receptor (CAR) T cells (auto-CARs). These successes have prompted studies of auto-CARs in second-line settings, in which axicabtagene ciloleucel and lisocabtagene maraleucel both showed improved event-free survival over autologous hematopoietic cell transplantation (AHCT). While encouraging, this data also highlights that 60% of patients relapse or progress following treatment with auto-CARs. Individual disease characteristics and logistical challenges of cell engineering also limit patients’ eligibility for auto-CARs. Allogeneic CAR T cells (allo-CARs) may address some of these limitations as they may mitigate delays associated with auto-CARs, thereby reducing the need for bridging chemotherapies and increasing availability of cellular products for patients with aggressive lymphomas. By being sourced from healthy donors who have never been exposed to cytotoxic chemotherapy, allo-CARs can be created from T cells with better fitness. Allo-CARs made from specific cellular subsets (e.g., stem cell memory or naïve/early memory T cells) may also have increased efficacy and long-term persistence. Additionally, allo-CARs have been successfully created from other cell types, including natural killer cells, gamma-delta T-cells and induced pluripotent stem cells. These cell types can be engineered to target viral antigens, enabling precision targeting of virally driven DLBCL. As allogeneic donor cells can be banked and cryopreserved in batches, they can be made more readily available, potentially reducing logistical hurdles and costs compared to engineering auto-CARs. This may ultimately create a more sustainable platform for cell therapies. Challenges with allo-CARs that will need to be addressed include graft versus host disease, alloimmunization, potentially decreased persistence relative to auto-CARs, and antigen escape. In short, the adaptability of allo-CARs makes them ideal for treating patients with r/r DLBCL who have progressed through standard chemotherapy, AHCT, or auto-CARs. Here, we review the published literature on patients with r/r DLBCL treated with allogeneic CAR products manufactured from various cell types as well as forthcoming allogeneic CAR technologies.

## Introduction

The treatment of r/r DLBCL is rapidly evolving as adoptive cellular technologies advance to the forefront. The impetus for their development was partially driven by the poor treatment options for this disease, demonstrated by the SCHOLAR-1 study in which only 26% of patients with r/r DLBCL treated with salvage chemotherapy achieved an objective response (OR), with a median survival of 6.3 months (1). Three auto-CAR products – axicabtagene ciloleucel (axi-cel), tisagenlecleucel (tisa-cel), and lisocabtagene maraleucel (liso-cel) – subsequently approved for r/r DLBCL achieved 40% complete remission (CR) rates at 1 year (2–4). Axi-cel (5) and liso-cel (6) both recently demonstrated improved event-free survival compared to autologous hematopoietic cell transplantation in the second-line setting for r/r DLBCL, which prompted the FDA approval of axi-cel as the first auto-CAR to move to the second line setting.

Despite these successes, auto-CARs are not curative in at least 60% of patients with r/r DLBCL. Limited long-term follow up data exists on patients who have received auto-CARs, but a recent report on 5-year outcomes from tisa-cel for r/r DLBCL demonstrated that the median duration of response was 61 months (7). Though 60% of patients had sustained responses, progression-free survival was only 31% at 5 years (7), indicating that a large fraction of these patients experienced disease relapse over time. Causes of auto-CAR failure are not yet well-elucidated, but multiple analyses suggest that tumor burden, need for bridging therapy, and poor performance status are associated with disease progression and decreased survival (8, 9). Studies of axi-cel and tisa-cel in France demonstrated that baseline disease burden (as measured by metabolic tumor volume) and presence of at least 2 areas of extranodal disease were predictive markers for early relapse or progression after auto-CAR (10). Similar data was reported in retrospective analyses of axi-cel in the United States (11). The need for bridging chemotherapy prior to auto-CAR also portended poor outcomes. In the TRANSCEND study, patients requiring bridging chemotherapy had decreased rates of CR and OR with liso-cel, and bridging itself was a high-risk feature similar to chemotherapy-refractory disease and older age (4). In the JULIET trial, 92% of patients received bridging therapy prior to tisa-cel and 30% of enrolled patients did not receive an infusion due to progression/death, reflecting the high-risk population in this study (3). An additional 7% did not receive an infusion due to manufacturing failures (3). Similar data was reported from 2^nd^-line auto-CAR studies in which bridging was permitted (6, 12). The need for bridging chemotherapy likely reflects more aggressive disease biology, as retrospective analyses have shown that patients who receive bridging have higher Eastern Cooperative Oncology Group Performance Status and International Prognostic Index scores, disease stage, bulky disease (> 10 cm), and higher lactate dehydrogenase levels at the start of therapy (13). Finally, the time to infusion of auto-CARs ranged from 17 to 54 days in the ZUMA-1, JULIET, and TRANSCEND trials (2–4), requiring that patients either have indolent enough disease to wait for manufacturing to take place or receive bridging therapy in the interim. Thus, a combination of disease factors and CAR product characteristics contribute to auto-CAR failures.

Allogeneic CAR therapies (allo-CARs) have the potential to address many of the above limitations. Advantages inherent to allo-CARs include the ability to optimize cell source, donor type, and manufacturing such that allo-CARs can be generated relatively rapidly, banked, and made readily available for patients who need urgent therapy. These qualities also should theoretically decrease production costs, thereby improving the long-term sustainability of adoptive cell therapy. Graft versus host disease (GVHD), alloimmunization, decreased long-term persistence, and antigen escape are major challenges to overcome for allo-CARs to become mainstays in the treatment of r/r DLBCL.

### Early Clinical Applications of Allo-CARs

Allo-CARs have thus far largely been used as donor lymphocyte infusions after allogeneic hematopoietic cell transplantation (allo-HCT) to either treat or mitigate relapse. Smith et al. previously reviewed clinical outcomes in patients who received either donor-derived allo-CARs or recipient-derived “pseudo-allo” CARs in the post-transplant setting (14). Among these trials, Kochenderfer et al. (15), Brudno et al. (16), Kebriaei et al. (17), and Lee et al. (18) enrolled patients with r/r DLBCL. Responses were mixed in these DLBCL cohorts: 3 patients achieved CR, 5 remained with stable disease, and 3 developed progressive disease. The time to cell production also differed between groups; Kochenderfer and Brudno et al. produced cells in 8 days (using retroviral transduction methods) (15, 16), Kebriaei et al. in 28 days (using a transposon/transposase system) (17), and Lee et al. in 11 days (using a simplified retroviral transduction system) (18, 19). Notably, no patients in these studies developed new GVHD, and 1 patient who had preexisting mild GVHD continued to have GVHD post-allo-CAR (**Table 1**) (16). Preclinical data suggests that the presence of CD19+ targets is protective against GVHD as tonic signaling through both the CAR and T cell receptor (TCR) promotes an exhaustion phenotype and subsequent apoptosis of alloreactive, GVHD-inducing cells (22). Despite this loss of alloreactive T cells, bulk CAR T cell populations contain other cells capable of attacking CD19+ tumors while sparing host tissue, which may explain why early trials of allo-CARs have not reported significant rates of GVHD (16, 22).

**Table 1:** Clinical Outcomes for Patients Treated with Allogeneic CAR Therapies.

CAR Type	Total number of Patients	Patients with r/r DLBCL	Acute GVHD	Chronic GVHD	Overall Efficacy (BOR)	Efficacy in r/r DLBCL (BOR)	T-cell Chimerism	Costimulatory Domain	Transduction Type
Axi-cel from allo-HCT donor (20)	7	7	3 (43%)	0 (0%)	3 CR, 1 PR (57%)	Same as overall	100% in 4/7, NR for others	CD28	Retrovirus
UCB-derived CAR-NK (21)	11	2	0 (0%)	0 (0%)	7 CR, 1 PR (73%)	1 CR (50%)	N/A	CD28	Retrovirus
EBV-CTL (41 )(NCT01430390)	10	1	1 (10%)	0 (0%)	7 CR (70%)	1 CR (100%)	NR	CD28	Retrovirus
Allo-HCT donor-derived CAR (16)	20	5	0 (0%)	2 (10%)	6 CR, 2 PR (40%)	1 CR, 3 SD (80%)	NR	CD28	Retrovirus
Allo-HCT donor-derived CAR (17)	19	2	2 (11%)	1 (5%)	11 CR (58%)	1 CR (50%)	NR	CD28	*Sleeping Beauty* transposon/transposase
Allo-HCT recipient-derived CAR (18)	21	1	0 (0%)	0 (0%)	3 CR (14%)	1 PD (0%)	NR	CD28	Retrovirus

Allo-HCT, allogeneic hematopoietic cell transplant; Axi-cel, axicabtagene ciloleucel; BOR, best overall response; CAR, chimeric antigen receptor; CR, complete remission; DLI, donor leukocyte infusion; EBV-CTL, EBV-specific cytotoxic lymphocytes; GVHD, graft-versus-host disease; NR, not reported; N/A, not available; PR, partial remission; r/r DLBCL, relapsed/refractory diffuse large B-cell lymphoma; SD, stable disease; UCB, umbilical cord blood.

More recently, donor-derived allo-CARs have been shown to induce responses in patients who previously received allo-HCT and auto-CAR therapy, raising the possibility of a graft-*vs*-lymphoma effect induced by the donor-derived allo-CARs (20). These early studies demonstrated the viability of allo-CARs as modified donor lymphocyte infusions for patients with otherwise refractory disease.

### Cellular Sources for Allo-CARs

Allo-CARs have been successfully generated from various cell types (**Table 2**), including those belonging to the innate immune system. Natural killer (NK) cells are a promising CAR candidate as they have intrinsic killing capabilities, do not cause GVHD, and are negatively regulated by major histocompatibility complex (MHC) class I molecules present on normal cells (39), which may limit off-target toxicities. They can also be derived from a variety of sources, including peripheral blood, NK cell lines, memory-like NK cells, human embryonic stem cells, CD34+ hematopoietic progenitor cells and induced pluripotent stem cells (iPSCs) (23). NK cells transduced with CARs (CAR-NKs) have demonstrated clinical efficacy; a Phase 1/2 trial of cord blood-derived CAR-NKs included 2 patients with r/r DLBCL, one of whom achieved a minimal residual disease (MRD)-negative CR for 15 months at data cutoff (21). No patients experienced cytokine release syndrome (CRS), immune effector cell-associated neurotoxicity (ICANS), or GVHD in this trial (21), providing proof-of-concept for decreased toxicity while maintaining anti-cancer effects. However, allogeneic NK cells are currently limited by lack of persistence in the absence of exogenous cytokine stimulation (40), decreased trafficking to tumor sites (41) and dysfunction induced by the immunosuppressive tumor microenvironment (39, 42, 43). Strategies to circumvent these obstacles include engineering CAR-NKs to express cytokine transgenes to improve persistence and chemokine receptors to improve homing capabilities (39). Either deleting or blocking checkpoint inhibitors on NK cells also may enhance their function.

**Table 2:** Advantages and Disadvantages of Potential Cellular Sources for Allogeneic CARs.

Cell Type	Advantages	Disadvantages
NK cells (21, 23)	Intrinsic killing capability, lack of GVHD, decreased risk of off-target toxicity due to MHC-1 mediated regulation	Decreased persistence and tumor trafficking
iNKT cells (24–26)	Lack of GVHD, can kill *via* CAR and TCR, CNS activity	Unknown persistence, function may be impaired by lymphodepleting chemotherapy, possibly more challenging to isolate due to rarity of T cell population
γδ T cells (27–30)	Capable of MHC-independent killing, lack of GVHD	Unknown persistence, variable transduction efficacy, some subsets may be immunosuppressive
iPSC (31, 32)	Unlimited replication potential; can facilitate scaling up of cell engineering and customization of antigen specificity	May cause GVHD due to TCR; if TCR removed, persistence may be reduced due to NK cell-mediated destruction
EBV-CTL (33–35)	Specific targeting of virally driven DLBCL, minimal risk of GVHD	Unknown persistence, potential for off-target toxicity on other infected tissues that express EBV antigens
SCM/early memory T cells (36–38)	Potential for improved engraftment and expansion with less exhaustion due to less-differentiated phenotype	Unknown persistence and cytotoxicity relative to more differentiated T-cell subsets

CNS, central nervous system; EBV-CTL, Epstein Barr virus cytotoxic T cell; iNKT, invariant natural killer/T; iPSC, induced pluripotent stem cell; MHC, major histocompatibility complex; NK, natural killer; SCM, stem cell memory; TCR, T cell receptor.

Invariant NK/T (iNKT) cells have recently shown promise as another platform for CAR engineering. These relatively rare cells co-express T and NK cell markers with a semi-invariant TCR that recognizes antigens presented on CD1d, an MHC class I-like molecule (24). CAR-modified iNKT cells were first developed to target the GD2 ganglioside on neuroblastoma in murine models, where they demonstrated antitumor activity without causing GVHD (44). Further murine and human studies have shown that iNKT cells actually suppress GVHD, likely through expansion of T regulatory cells (24). When transduced with CD19 CARs, iNKT cells showed activity against B cell malignancies through both CAR-CD19 and CD1d-invariant TCR interactions, resulting in elimination of tumors in mice (including those with intracranial and relapsed lymphomas) (25). Subsequently, in a murine model of CD19+ lymphoma, Simonetta et al. showed that allogeneic CAR-iNKT cells exerted anti-tumor activity through cross-priming of CD8+ T cells, and were more effective at tumor control than conventional CAR T cells in the presence of host lymphocytes (26). This cross-talk between CAR-iNKT cells and CD8 T cells appears essential to CAR-iNKT function, which may be impaired by the typical lymphodepleting chemotherapy given prior to conventional auto-CAR therapy. Nonetheless, iNKT cells do not cause GVHD, can be easily expanded *ex vivo*, and utilize dual targeting mechanisms to eliminate tumors. These characteristics make iNKT cells excellent candidates for future CAR development strategies.

Like iNKT cells, γδ T cells participate in both innate and adaptive immunity and have cytotoxic mechanisms independent of the TCR-MHC interaction. γδ T cells recognize peptide antigens in an MHC-unrestricted fashion and can express ligands such as NK receptors and toll-like receptors to identify and destroy target cells damaged by infection, malignancy, or other stressors (27). Their multimodal approach to cell destruction makes γδ T cells good candidates for CAR engineering. An anti-CD20 allo-CAR made from γδ T cells has demonstrated manufacturing feasibility and improved cytotoxic activity over conventional CAR-T cells in a preclinical model of B-cell lymphoma (28), which has subsequently led to a Phase 1 trial in humans (NCT04735471).

The current dearth of easily available, antigen-specific T-cells could also be addressed by using iPSCs as CAR platforms as they have nearly unlimited replication potential. Themeli et al. demonstrated the feasibility of this approach by generating iPSC clones from peripheral blood lymphocytes using dedifferentiation methods, transducing a clone with a CD19-targeted 2^nd^-generation CAR, and directing the differentiation of the CAR-iPSC clone towards T-lymphoid lineages (31). This method produced functional iPSC-derived CAR T-cells that expanded *ex vivo* and induced complete tumor regression in murine models, albeit more slowly than standard αβ or γδ CAR T cells (31). Despite retaining the αβ-TCR, the iPSC-derived CARs also exhibited phenotypic and functional characteristics typical of γδ T cells (31). The main limiting factor for iPSC-derived allo-CARs is the potential for GVHD due to HLA mismatches and endogenous TCR expression (32). Genome editing techniques could be employed to remove these proteins from allo-CARs, but this would potentially make them susceptible to NK cell-mediated destruction due to lack of MHC I expression (32).

Epstein-Barr virus accounts for 20% of DLBCL cases (33), making this virus an important therapeutic target. EBV-driven lymphoproliferative diseases (EBV-LPDs) are categorized as a DLBCL subtype that develops after allo-HCT and solid organ transplantation. EBV-LPDs have been shown to be amenable to T-cell therapies targeting EBV latent membrane proteins (LMPs). In one early study of autologous cytotoxic T cells, three out of 4 patients with r/r DLBCL achieved CR (34). Subsequently, donor-derived T-cells engineered to target LMPs were evaluated in patients after allo-HCT for r/r lymphoma and chronic lymphocytic leukemia (CLL). Three of the 19 patients in this study had r/r DLBCL and an additional 2 had EBV-LPDs. All 5 remained in CR for 8 weeks post-infusion, and 3 of these patients maintained a CR for over 3 years (33). Reactivation of GVHD was seen in 2 of the patients with DLBCL, one of whom died of allo-HCT-related complications 6 months post-infusion (33). Patients with B-cell diseases had an 80% overall survival at 2 years, demonstrating the safety and effectiveness of engineered allogeneic T-cells as adjuvant therapy for virally driven lymphomas. More recently, the EBV-specific allogeneic T-cell therapy tabelecleucel (which employs TCR-targeting and HLA restrictions to specifically attack EBV-transformed cells) (45) induced CRs in 32/76 patients (42%) with r/r EBV+ LPDs (46). The overall response rate (complete and partial remissions) in this trial was 63%, with 91% overall survival at 1 year and 86% at 2 years without any GVHD, CRS, neurotoxicity, or graft rejection (46). EBV-specific allo-CAR T-cells have also been developed and tested in the consolidation setting after AHCT for non-Hodgkin lymphomas. A Phase 1 trial demonstrated 100% CR rates in 4 such patients, with a median overall survival of 30.8 months and no CRS or neurotoxicity (35). This encouraging early data suggests that EBV+ lymphomas are attractive disease candidates for future allo-CAR trials.

### The Role of Source Cell Fitness in CAR Efficacy

In studies of DLBCL relapses after axi-cel, suboptimal T-cell fitness (measured by prolonged cell doubling time) was associated with treatment resistance (47). Prior chemotherapy exposure likely plays a role in decreasing T-cell fitness, as laboratory evidence suggests that cyclophosphamide, doxorubicin, and cytarabine impact mitochondrial function and induce lingering deficits that impair *ex vivo* stimulation (48). Studies of T-cell expansion in children undergoing chemotherapy for various malignancies demonstrated that T-cells from children with non-Hodgkin’s lymphoma had among the lowest post-chemotherapy expansion potentials (36). Another factor that impacts T-cell fitness is the phenotype of the cells comprising the CAR product. Preclinical evidence suggests that less-differentiated (CD62L+) T-cell populations are capable of enhanced engraftment, expansion, and persistence relative to more differentiated effector subsets (37). CCR7^+^CD45RA^+^ cells have a stem-like central memory (SCM) phenotype, and a decreased frequency of these cells relative to tumor burden has been associated with increased CAR-T doubling times and failure to achieve durable responses in patients with DLBCL (47). Enriching for SCM T-cells in CAR products may thus improve response rates. Further data supporting this hypothesis comes from transcriptomic analysis of CAR-T cells used for chronic lymphocytic leukemia, which identified a subpopulation of CD27^+^CD45RO^low^ CAR-T cells that were seen more consistently in patients with complete responses (38). These cells were then shown to proliferate extensively *ex vivo* while maintaining a less differentiated state (38). A Phase 1 trial using central memory-enriched CAR T-cells (albeit after AHCT) for r/r DLBCL demonstrated the safety and feasibility of this approach, and 5 of 11 patients maintained their best responses (either PR or CR) at 1 year post-therapy (49). CAR products engineered with defined 1:1 CD4:CD8 ratios have also shown improved antitumor activity relative to unselected T-cells in mouse models (50) and have demonstrated efficacy in patients with r/r DLBCL (51).

### Challenges With Allo-CARs

As alluded to in prior sections, the key obstacles that allo-CARs will need to overcome to become more broadly used include GVHD, allo-CAR rejection (i.e., alloimmunization), and potentially decreased long-term persistence. Though not specific to allo-CARs, resistance mechanisms such as antigen escape will also need to be addressed to maximize allo-CAR effectiveness.

GVHD and alloimmunization both reflect the challenge of addressing HLA-incompatibility between the allo-CAR donors and patients. Overall, early clinical data with allo-CARs revealed encouragingly low rates of GVHD (15, 16, 18), though innovations in allo-CAR engineering are aiming to lower that risk even further. GVHD is thought to be mediated through the interaction of donor αβ-TCR with host MHC complexes; therefore, strategies to abrogate GVHD thus far have involved elimination of the αβ-TCR or the use of non-alloreactive cells (such as NK, iPSCs, and virus-specific T-cells discussed above) (52). Several genome editing techniques are capable of creating insertions or deletions at the site of the TCR alpha constant (TRAC) locus, causing disruption of this gene and the inability to form the TCR complex (53, 54). Another technique is to use a recombinant adeno-associated virus or lentivirus to insert the CAR transgene directly into the TRAC locus, thereby replacing the TCR with a CAR construct and enabling homogenous and uniform levels of CAR expression (54). Generating allo-CARs from less-differentiated T-cell subsets (e.g., SCM T cells) may also reduce GVHD risk as these cells have limited TCR specificity (55, 56).

Alloimmunization is expected to be a significant issue with allo-CARs. Though lymphodepletion is necessary for expansion of infused cells, immune recovery with time is expected to result in rejection of HLA-mismatched cells. Preexisting donor-specific antibodies (e.g., from prior pregnancies or blood product transfusions) may also mediate rejection (57). Alemtuzumab has been used to further deplete host T-cells prior to allo-CAR therapy, though this increases the risk of subsequent cytopenias and infections (58). One potential way to minimize the risk of rejection is to bank T cells that match key HLA alleles (HLA-A, HLA-B, and HLA-DR) with most of the population, as has been done with solid organ and umbilical cord transplantation.

CAR T-cell persistence is a multifactorial issue that has yet to be fully explained. A prior review of auto-CAR resistance mechanisms (59) identified several contributors, including initial T cell quality (18, 60), phenotype (38, 61–63), and choice of costimulatory domain (2, 3). In a preclinical model, T cell quality was assessed by comparing the viability and phenotypes of CAR T cells created from young (6-12 weeks) and aged (72 weeks) mice. T cells derived from the older mice were more cytotoxic but were shorter-lived and had a less memory-like phenotype than cells from younger mice (64). Phenotype was also studied in a retrospective analysis of a Phase 1 trial evaluating CAR T cells for leukemia and lymphoma in children and young adults. In this study, CAR T cells that initially had greater expression of PD-1 and LAG-3 and decreased expression of TNF-α were associated with dysfunctional responses and decreased cytokine production in response to stimulation (65). Dysfunctional response was a clinically meaningful outcome as it was defined as either the inability to achieve remission or disease progression after remission but before CAR engraftment (65). Finally, costimulatory domains also play a significant role in CAR persistence; in the landmark study of tisa-cel in children and young adults with B-cell leukemias, 4-1BB-based auto-CARs persisted for a median of 168 days compared to 30 days for CD28-based auto-CARs (66). Whether CAR persistence equates to improved remission duration in r/r DLBCL is unclear and needs further investigation.

Antigen escape is a common CAR resistance mechanism observed with leukemias (59), though this phenomenon has also been reported in lymphomas. One case report demonstrated sequential loss of CD19 and CD22 over time in a pediatric patient with DLBCL treated with auto-CARs targeting those antigens (67). The patient also had a homozygous TP53 mutation, which the authors hypothesized may have driven clonal expansion of CD19- and CD22-negative cells (67). A similar case was previously reported demonstrating absent surface CD19 expression (with conserved cytoplasmic expression) in primary mediastinal B-cell lymphoma (PMBCL) treated with auto-CARs (68). In this case, missense mutations in exon 4 of the CD19 gene and deficiencies in DNA mismatch repair proteins were identified in PMBCL clones that were seen pre-treatment and ultimately became dominant post-treatment (68). More recently, CD19 exon 3 point mutations in a case of high grade B-cell lymphoma were found to confer resistance to certain subsets of auto-CAR populations while retaining sensitivity to others (69). These cases are illustrative of how mutations impact loss/alteration of CAR surface targets, presenting an important area for further research.

Numerous methods are under investigation to overcome antigen escape. Multi-antigen-targeted CARs (e.g., bispecific CD19/22) have been shown to induce durable remissions in CD19-low or negative disease previously treated with anti-CD19 auto-CARs (70). Studies of Bryostatin1, which can improve CAR functionality and durability by increasing expression of CD22 on DLBCL cell lines, have demonstrated the feasibility of modulating target antigen density in lymphomas (71, 72). Modifying the CAR construct can also increase affinity for lower antigen density tumors. Majzner et al. demonstrated that CD28 endodomain-containing CARs are better able to kill and proliferate in response to low antigen density cells *in vitro* compared to their counterparts with 4–1BB endodomains (73). Alterations to the hinge transmembrane domains or CAR zeta chains also can improve recognition of low density antigens (73). These findings will undoubtedly influence how allo-CARs will be engineered in the future.

## Discussion

This review attempts to illustrate the features that make allo-CARs appealing alternatives to auto-CARs. Considering that many auto-CAR patients currently receive 3 or more lines of therapy prior to cell collection, it is likely that their CARs are made from less fit T cells at baseline. Less-differentiated T-cells are also relatively less abundant in circulation (37) and naïve and memory T-cell subsets also differ widely among patients with lymphoma (51), which can add to the challenge of engineering quality auto-CAR products in a timely fashion. Improvements will undoubtedly be made to manufacturing processes to shorten time-to-infusion, but patients with aggressive disease requiring treatment within days to a week are limited in their ability to successfully receive auto-CARs. Using healthy donors to create allo-CARs will alleviate many of these burdens. Allogeneic donors will not be cytopenic at baseline, allowing for multiple CAR products to be made from a single apheresis and eliminating the possibility of manufacturing failures related to insufficient cell collection. Obtaining T-cells from healthy donors also facilitates cell banking as collected cells will be cryopreserved in batches (74), enhancing the ability to rapidly engineer and standardize allo-CARs for different patients, potentially even in an HLA-matched fashion (74). This will particularly benefit patients with aggressive malignancies like r/r DLBCL who cannot afford to wait for therapy. There may also be theoretical cost advantages to allo-CARs due to improved efficiencies in scaling and manufacturing, though empiric evidence for this is not available yet. Ultimately, the adaptability of allo-CARs will enable their success and longevity in the world of adoptive cell therapies.

## Author Contributions

NJ and MS together developed the conceptual ideas behind this project. NJ wrote the manuscript with assistance and editorial support by MS. All authors contributed to the article and approved the submitted version.

## Funding

MS is supported by the following funding sources: Burroughs Wellcome Fund Postdoctoral Enrichment Program, American Society of Hematology-Robert Wood Johnson Foundation Harold Amos Medical Faculty Development Program, NHLBI K08 HL156082-01A1 and V Scholar Grant for Black/African American Cancer Researchers.

## Conflict of Interest

The authors declare that the research was conducted in the absence of any commercial or financial relationships that could be construed as a potential conflict of interest.

## Publisher’s Note

All claims expressed in this article are solely those of the authors and do not necessarily represent those of their affiliated organizations, or those of the publisher, the editors and the reviewers. Any product that may be evaluated in this article, or claim that may be made by its manufacturer, is not guaranteed or endorsed by the publisher.
